# F105. MEASURING EMPATHY IN SCHIZOPHRENIA: THE EMPATHIC ACCURACY TASK AND ITS CORRELATION WITH OTHER EMPATHY MEASURES

**DOI:** 10.1093/schbul/sby017.636

**Published:** 2018-04-01

**Authors:** Rozanne van Donkersgoed, Bouwina Sportel, Steven De Jong, Marije aan het Rot, Alexander Wunderink, Paul Lysaker, Ilanit Hasson-Ohayon, Andre Aleman, Marieke (Gerdina) Pijnenborg

**Affiliations:** 1 GGZ Friesland, Rijksuniversiteit Groningen; 2 Department of Psychotic Disorders Assen; 3 Rijksuniversiteit Groningen; 4 Friesland Mental Health Services; 5 Roudebush VA Medical Center, Indiana University School of Medicine; 6 Bar-Ilan University; 7 University Medical Center Groningen

## Abstract

**Background:**

Empathy is a complex interpersonal process thought to be impaired in individuals with schizophrenia spectrum disorders. Past studies have mainly used questionnaires or performance-based tasks with static cues to measure cognitive and affective empathy. In contrast, we used an Empathic Accuracy Task (EAT) designed to capture the more dynamic aspects of empathy by using video clips in which perceivers continuously judge emotionally charged stories of various targets. We compared individuals with schizophrenia to healthy controls on the EAT and assessed correlations among the EAT and three other commonly used empathy tasks.

**Methods:**

Patients (n=92) and healthy controls (n=42) matched for age and education, completed the EAT, the Interpersonal Reactivity Index, the Questionnaire of Cognitive and Affective Empathy and the Faux Pas task. Differences between groups were analyzed and correlations were calculated between empathy measurement instruments.

**Results:**

The groups differed in EAT performance, with controls outperforming patients. A moderating effect was found for the emotional expressivity of the target: while both patients and controls scored low when judging targets with low expressivity, controls performed better than patients with more expressive targets. Though there were also group differences on the cognitive and affective empathy questionnaires (with lower scores for patients in comparison to controls), EAT performance did not correlate with questionnaire scores. Reduced empathy performance did not seem to be part of a generalized cognitive deficit, as differences between patients and controls on general cognition was not significant.

**Discussion:**

Individuals with schizophrenia benefit less from the emotional expressivity of other people than controls, which contributes to their impaired empathic accuracy. The lack of correlation between the EAT and the questionnaires suggests a distinction between self-report empathy and actual empathy performance. To explore empathic difficulties in real life, it is important to use instruments that take the interpersonal perspective into account.

